# Musical groove listening does not enhance primary motor cortex activation

**DOI:** 10.1162/IMAG.a.1185

**Published:** 2026-04-03

**Authors:** Samantha R. O’Connell, Grace E. Wilson, Dan H. Berkowitz, Erin E. Hannon, Joel S. Snyder

**Affiliations:** Department of Psychology, University of Nevada, Las Vegas, Las Vegas, NV, United States; Department of Psychology, Wesleyan University, Middletown, CT, United States

**Keywords:** groove, music, primary motor cortex, event-related potentials

## Abstract

Groove is the pleasurable urge to move to the beat of music, a universally experienced human phenomenon. Because listeners are often inspired to move to music with groove, we asked whether high-groove music would promote greater motor system activity than low-groove music, noise, or silence, as shown by shorter latency and/or greater amplitude of the lateralized readiness potential (LRP), an event-related potential measure of neural response preparation and execution in the primary motor cortex. In Experiment 1, 21 participants were presented with 1 of 2 symbols (@ and #) on a computer monitor and pressed a button with their left or right hand, depending on which symbol was presented, while listening to high-groove music, low-groove music, noise, or silence. Throughout the duration of the experiment, electroencephalography was recorded at the scalp and we calculated stimulus-locked and response-locked activity by averaging across correctly responded, non-artifact trials. In Experiment 2, 31 participants were tested with the same stimuli, procedure, and analysis, with the following exception: instead of responding to visual stimuli, participants pressed a button with their left or right hand, depending on whether they heard an occasional increase or decrease in intensity in the music or noise. None of the analyses in either experiment showed differences in LRP latency or amplitude between the high-groove or low-groove conditions. Several of the analyses yielded Bayes Factor values greater than 3 in favor of the null hypothesis. Thus, we found no evidence that high-groove music enhances activity in the primary motor cortex. Future studies should use other brain activity measurement and manipulation techniques to test the generality of our findings, including techniques that can separately measure different motor and auditory cortical areas.

## 1 Introduction

Music and movement are fundamentally intertwined. Until the advent of electronic music technologies, performing music required movement of the vocal tract in the case of singing and or movement of the fingers and limbs in the case of stringed instruments and drums. Dance and foot-tapping in response to music are widespread examples of the strong connection between music and movement, as are the near-universal rhythmic aspects of music that enable such synchronized movement (Savage et al., 2015). Furthermore, two recent theories of music evolution propose that music may have evolved at least in part to facilitate synchronized movement for purposes of signaling group strength or for social bonding, respectively (Mehr et al., 2021; Savage et al., 2021). Even very young children (Fujii et al., 2014; Kim & Schachner, 2023; Kragness et al., 2022; Zentner & Eerola, 2010) and non-human animals (Schachner et al., 2009; Patel et al., 2009) spontaneously move to music, though the accuracy and precision with which they synchronize are typically less than with adult humans.

*Groove* is the subjective wanting to move to music, often as a result of listening to music with a clear sense of beat or meter (Janata et al., 2012; Madison, 2006; Pressing, 2002; for a review, see Stupacher et al., 2022), and is associated with liking and familiarity of the music (Janata et al., 2012; Matthews et al., 2023; Senn et al., 2021, 2023). *Beat* perception refers to the experience of temporally regular salient time points in the music that guide movement, such as foot-tapping, dancing, and coordination with other musicians (for a review of research on beat, meter, and groove, see Snyder et al., 2024). *Meter* perception occurs when multiple beat levels are simultaneously experienced, with coinciding beats from the multiple levels leading to perception of a hierarchy of strong and weak beats, as in the repeating strong-weak-weak pattern of a waltz. One of the best studied aspects of music that influences perception of groove is *syncopation*, which occurs when rhythmic patterns have events that occasionally occur on relatively weak beats. This can make tapping to music more challenging (Snyder & Krumhansl, 2001; Toiviainen & Snyder, 2003), but it does not necessarily disrupt perception of the beat or meter. In fact, moderate syncopation might enhance groove, as suggested by the observed inverted U-shaped relationship between syncopation and groove, with listeners preferring not too much and not too little syncopation (Witek et al., 2014; Matthews et al., 2019). Groove can also be influenced by other features of music besides syncopation that add to perceived complexity of the music, for example, harmonic complexity, spectral flux, tempo/note density, multi-modal input, genre of music, and the variety of instruments used (Cameron et al., 2022; Janata et al., 2012; Hove et al., 2020; Matthews et al., 2019; Seeberg et al., 2025; Senn et al., 2016, 2018, 2019; Sioros et al., 2022; Stupacher et al., 2016; Wesolowski & Hofmann, 2016). In addition, characteristics of the listener may influence or at least correlate with groove ratings, for example, the amount of music and dance training, and several aspects of music perception skill (Fink et al., 2022; Matthews et al., 2019; O’Connell et al., 2022).

Clearly, we know much about the features of music and aspects of the listener that lead to a stronger sense of groove, but we still have limited understanding of the neural basis of groove. A very general mechanistic theory of groove is that it arises from activation of motor areas of the brain (Janata et al., 2012; Stupacher et al., 2013). Theories of groove propose that the experience arises in part from interactions between premotor cortex and auditory cortex, due to either predictive coding mechanisms (Vuust & Witek, 2014; Vuust et al., 2018) or oscillatory entrainment mechanisms (Zalta et al., 2024).

Indirect support for these theories comes from functional magnetic resonance imaging (fMRI) studies of beat and rhythm perception, which often show activation in areas linked to motor processing, such as the premotor cortex, supplementary motor areas, basal ganglia, and the cerebellum (Araneda et al., 2017; Chen et al., 2008a, 2008b; Grahn & Brett, 2007; Grahn & McAuley, 2009; Grahn & Rowe, 2009; Li et al., 2019; Sakai et al., 1999; Schubotz & von Cramon, 2001; Schubotz et al., 2000). Similarly, studies of beat perception using electroencephalography (EEG) and magnetoencephalography (MEG) have repeatedly found beta activity, which is strongly associated with motor processing (Comstock et al., 2021; Fujioka et al., 2010, 2012, 2015; Iversen et al., 2009; Snyder & Large, 2005). These investigations provide strong support for the engagement of motor planning regions during music listening; however, very few provide evidence for the engagement of primary motor cortex, the cortical region most responsible for the direct activation of skeletal muscles during volitional movement.

Studies directly linking groove to brain activity are more rare, but one fMRI study found activity in both motor-related and reward-related parts of the basal ganglia (i.e., dorsal striatum and ventral striatum) and the supplementary motor area that was greater in response to moderate complexity rhythms (Matthews et al., 2020). A recent MEG study provided evidence that activity in auditory-motor cortical pathways correlates with the experience of groove (Zalta et al., 2024). Finally, a transcranial magnetic stimulation (TMS) study provided some of the first evidence for greater primary motor activation while listening to high-groove music than while listening to low-groove music (Stupacher et al., 2013). In particular, this study demonstrated motor cortical modulation during passive music listening and did not require participants to attend to the rhythm of the music or perform a task to report their experience of groove.

The current study seeks to provide converging evidence with the latter TMS study, by measuring EEG activity that comes from primary motor cortex while participants listen to music that is perceived as high in groove or low in groove. Like the Stupacher et al. (2013) study, we aimed to investigate the naturalistic experience of groove and, therefore, used popular and commercially available music. Using EEG provided a way to investigate these questions without interrupting with listening experience with TMS pulses to the scalp. We also included control conditions in which participants performed motor tasks while listening to noise that was spectrally matched to the music, or in silence.

The specific brain response we measured is an event-related potential (ERP) called the lateralized readiness potential (LRP). Originally described by Kornhuber and Deecke (1965) as the *bereitschaftspotential*, the LRP is among the most studied ERPs related to motor preparation and execution (Smulders & Miller, 2011). It is usually observed during a visual choice task in which participants respond to one stimulus by pressing a button with the right hand and to another stimulus by pressing a button with the left hand. The LRP is known to arise from primary motor cortex starting 100–200 ms before the execution of a choice response (De Jong et al., 1990; Leuthold & Jentzsch, 2002). By examining both stimulus-locked and response-locked LRPs, it is possible to assess the neural activity of motor preparation and motor execution, respectively. Thus, this ERP can indicate preparatory motor activity that precedes the physical response (Kappenman et al., 2016; Smulders & Miller, 2011), consistent with evidence from single-neuron studies showing that primary motor cortex is modulated during both motor preparation and motor execution (Shenoy et al., 2013). While targeting of premotor cortical areas might also be informative as shown by recent studies on groove (Matthews et al., 2020; Spiech et al., 2025; Zalta et al., 2024), it is reasonable to assume that effects of groove on premotor cortex would also influence activity in primary motor cortex due to the highly interconnected nature of these brain areas.

Experiment 1 used a conventional LRP paradigm with visual stimuli that required responses with the right or left hand while participants heard in the background high-groove music, low-groove music, noise that was spectrally matched to the music, or silence. We calculated LRPs as the difference between waveforms from electrodes C3 and C4, as is typical in LRP studies (e.g., Kappenman et al., 2016). Because most individuals are compelled to move to music with groove, we predicted that listening to high-groove music, compared with listening to low-groove, noise, or silence, would elicit shorter LRP latencies and larger LRP amplitudes. In particular, for both the stimulus-locked and response-locked LRPs, we expected shorter LRP latencies and larger LRP amplitudes in the high-groove condition than in the low-groove condition, which would indicate facilitation of motor processing for high-groove music (Kappenman et al., 2016). In case listening to any kind of music enhanced activity in motor cortex (regardless of groove condition), we included a condition in which participants listened to noise with similar spectral energy as the music, and in case any type of sound enhanced motor cortical activity, we included a condition with no sound at all.

The results we present below from Experiment 1 failed to show any LRP differences between conditions. We attributed the lack of significant results to the visual stimuli used during the EEG experiment which may have diverted attention away from the music and toward the button-pressing task. We, therefore, conducted Experiment 2 to test whether differences between the high-groove and low-groove conditions in LRP amplitude required active attention to the music. Experiment 2 presented the same sound conditions (high-groove, low-groove, noise, and silence) but used auditory stimuli—delivered as brief changes in sound intensity embedded into the music—to cue button presses to channel attention toward the music. The hypotheses for Experiment 2 were the same as those listed above for Experiment 1, namely shorter LRP latencies and larger LRP amplitudes for the high-groove condition than for the low-groove condition for both the stimulus-locked and response-locked LRPs.

## 2 Experiment 1

### 2.1 Methods

#### 2.1.1 Participants

Both experimental procedures were approved by the University of Nevada Las Vegas’ (UNLV) Institutional Review Board (protocol #0710-2518). All participants were UNLV undergraduate and graduate students recruited using UNLV’s SONA participant pool. Participants received course credit upon completion of the study. Prior to testing, participants were asked to complete a questionnaire informing us of their demographics; history of learning, neurological, or motor disorders; musical experience; dance experience; language experience; and listening preferences. All participants received a hearing screening prior to participation. Initial exclusionary criteria included a history of a learning, neurological, or motor disorder, and an audiogram with >25 dB HL for any pure tone frequencies from 500 to 8000 Hz. Post-testing exclusionary criteria included participants who did not complete the study and EEG data with an artifact rejection rate of >30%.

For Experiment 1, 28 normal hearing adults (<25 dB HL pure tone frequencies from 500 to 8000 Hz) were originally recruited for Experiment 1. Three participants were excluded due to a history of traumatic brain injury resulting in a concussion or a subdural hematoma; 1 participant was excluded due to study incompletion; and 3 participants were excluded for EEG data with an artifact rejection rate of >30%. The final 21 participants were between the ages of 18 and 30 years (*M* = 21.76 years, *SD* = 4.073 years) and had no history of learning, neurological, and motor disorders. All but one participant were right handed. Fifteen participants self-reported having musical experience (of those people, age start: *M* = 10.33 years old, *SD* = 3.086 years old, range = 5–13 years old; years of practice: *M* = 5.00 years, *SD* = 4.520 years, range = 1–16 years) and 12 participants self-reported having dance experience (of those people, age start: *M* = 8.67 years old, *SD* = 4.637 years old, range = 3–16 years old; mean years of practice: *M* = 5.11 years, *SD* = 8.343 years, range = 1–27 years). Of these participants, eight had both music and dance experience.

#### 2.1.2 Stimuli

Twenty songs were selected from the Janata et al. (2012) music library (see Table 1 for a complete list) and used in both experiments. Ten were high-groove songs and 10 were low-groove songs. Groove category was determined by the ratings reported in Experiment 1 of Janata et al. (2012). All selected songs were performed in a 4/4 time signature. Because tempo and instrumentation influence groove perception (Janata et al., 2012), five high-groove and five low-groove songs were matched based on tempo, instrumentation, vocals, and meter. Four out of the five high-groove/low-groove matched pairs were selected from those used in Stupacher et al. (2013). Ideally, all 20 songs would have been grouped into 10 high-groove/low-groove matched pairs; however, given the desire to use the most highly rated and lowly rated songs and that high-groove and low-groove songs are of different genres (e.g., high-groove songs are of mostly the “soul” genre while low-groove songs are mostly of the “rock” and “folk” genres), it was impossible to create matched pairs for all songs in this library. Using the software Audacity 2.1.2, stimuli were truncated to 15-second (behavioral) and 65-second (EEG) segments and normalized to be the same volume. Like what was used in Janata et al. (2012), song stimuli were segmented based on what is presented in the iTunes song preview, starting at ~45 seconds into the song.

**Table 1.** Songs used in practice and experimental trials.

Song name	Artist	Groove category	Genre	Janata et al. (2012) groove rating	BPM
Superstition	Stevie Wonder	High	Soul	108.7	96
Lady Marmalade	Patti LaBelle	High	Soul	102.5	112
It’s a Wrap (Bye, Bye)	FHI (Funky Hobo #1)	High	Soul	105.9	96
Flash Light	Parliament	High	Soul	105.1	108
Mama Cita	Funk Squad	High	Soul	101.6	96
Sing, Sing, Sing	Benny Goodman	High	Jazz	97.4	124
Look-Ka Py Py	The Meters	High	Soul	92.5	175
Bring the Funk	Ben Harper	High	Soul	89.9	104
I Used to Love Someone	Anthony Hamilton	High	Soul	88.7	92
Bad Tune	Earth, Wind, and Fire	High	Soul	86.2	120
Cheeseburger in Paradise	Jimmy Buffet	Low	Rock	48.6	140
Orion’s Belt	The String Cheese Incident	Low	Rock	47.9	140
Comfortably Numb	Pink Floyd	Low	Rock	42.3	64
Bryter Layter	Nick Drake	Low	Folk	40.4	128
Better Man	Pearl Jam	Low	Rock	39.8	124
Space Oddity	David Bowie	Low	Rock	38.7	140
Ray Dawn Balloon	Trey Anastasio	Low	Rock	38.5	81
Flandyke Shore	The Albion Band	Low	Folk	36.5	92
Beauty of the Sea	The Gabe Dixon Band	Low	Rock	32.1	113
Sweet Thing	Alison Brown	Low	Folk	30.9	96
Must Be Dreaming	Frou Frou	Mid	Rock	60.9	148

For noise conditions, spectrally matched noise tracks were generated in the MATLAB 2016b programming environment (The Mathworks, Natick, MA, USA). For each groove category (high/low), 65-second .wav files of all 10 songs were averaged to generate a grand average wave file. Then, using the function *fft*, the fast Fourier transform (FFT) of the grand average wave file was taken to obtain the frequency information. Next, the envelope of the FFT was taken using the function *envelope.* To make a noise file, white Gaussian noise was generated using the *wgn* function, then was transposed into the frequency domain by taking the FFT. To match the frequency envelope of the grand average song to the white noise, the grand average envelope was multiplied by each component of the white noise FFT matrix (grand average envelope * white noise FFT). To transpose the white noise back into the temporal domain, the inverse FFT was taken (*ifft*). Each mono channel was scaled to its maximum amplitude to ensure equal stereo scaling. The two generated spectrally matched noise files (high groove and low groove) were then imported into Audacity and normalized to match the intensity of the individual songs. A silent file was generated and truncated to a 65-second clip in Audacity. Visual and auditory stimuli were presented using Presentation (Neurobehavioral Systems, Inc., Albany, CA) software on a Dell 22-inch LCD monitor (Dell Technologies, Round Rock, TX, USA) using EA-3 ear inserts (Etymotic Research, Elk Grove Village, IL).

The symbols “@” and “#” were presented for 200 ms as visual stimuli to indicate to the participant when to press the response button and with which hand. Stimuli were presented on the center of the screen in white on a black background measuring at a visual angle of 3.4095 degrees. In between response trials, a fixation cross, also in white on a black background (measuring at this visual angle), was visible to limit lateral eye movements. The monitor was viewed at a distance of 42 inches.

#### 2.1.3 EEG setup

EEG data were collected in an IAC single-walled sound-attenuated chamber using Ag/AgCl electrodes from a Biosemi ActiveTwo 72-electrode system (Biosemi, Amsterdam, Netherlands), with 64 electrodes placed on the scalp according to the International 10/20 system. Eight additional electrodes were placed on more inferior locations on the face and head: eye movements were monitored using electrodes on the outer canthi and on the inferior and superior areas of the left orbit, and additional electrodes were placed on the left and right mastoids, and 1/2 centimeter in front of the preauricular point of the left and right ear. Responses were recorded in LabView 2.0 (National Instruments, Austin, TX), digitized at 1024 Hz, and collected with an online bandpass filter of 0.1 to 500 Hz. Offset voltage was <25 mV for all electrodes.

#### 2.1.4 Procedure

Presentation software (Neurobehavioral Systems, Inc., Albany, CA) was used to program this experiment. Participants listened to sound or silence in the background while performing a primary visuo-motor task of responding with left- and right-hand button presses to the symbol “@” or the symbol “#”. The experiment began with two practice trials to ensure participants were performing the task correctly. Practice trial 1 was presented in silence. Practice trial 2 was performed while concurrently listening to “Must Be Dreaming” by Frou Frou, a mid-groove song, as categorized by Janata et al. (2012). During practice, a mid-groove song was preferred to a high- or low-groove song to prevent unbalanced exposure to a particular auditory stimulus category prior to testing. For each practice trial, participants were asked to make left- and right-hand button presses to either the symbol “@” or the symbol “#” using the bottom two buttons on the Cedrus RB-830 as quickly and as accurately as possible. Responses in the two practice trials were counterbalanced to rehearse both stimulus-to-response mapping orientations. For example, if practice trial 1 assigned left-hand button presses to the symbol “@”, the left-hand button presses for practice trial 2 were assigned to the symbol “#”. The duration of each practice trial was 65 seconds: the first 5 seconds was used as auditory preparation for the trial followed by 60 seconds of active button presses. Audio tracks were played for the entire duration of a trial. Each visual stimulus was followed by a blank inter-stimulus interval (ISI) of either 1100, 1200, 1300, or 1400 ms presented randomly with an even distribution. No single ISI was presented twice in a row. The fixation cross was shown during the ISI. For each practice trial, a total of 40 stimuli were presented, 20 for each hand. Hand choice was also presented randomly. In order to continue onto practice trial 2, participants needed to correctly respond to > 80% of the stimuli (>32/40 stimuli). If they failed to complete practice trial 1 with at least 80% correct trials, they were asked to repeat practice trial 1. Participants were asked to not move during the experiment, except to make the requested button presses.

Forty total experimental trials were presented in 4 blocks of 10 trials each, each trial containing 40 cued button presses. Ten high-groove song trials, 10 low-groove song trials, 10 spectrally matched noise track trials (5 high-groove, 5 low-groove), and 10 silence track trials were played in a quasi-random order over the 40 trials (i.e., no two audio tracks of the same category were played in a row). Within each block, no more than three audio tracks of the same category were presented. The task mimicked what was presented in the practice trials. The inter-trial interval (ITI) was 5 seconds. After the completion of each block, a participant-determined break occurred before proceeding onto the next block. Each block had a duration of 11.58 minutes, equaling a total experiment duration of 46.33 minutes, plus participant-determined breaks. For each auditory category, 400 left- and right-handed responses were collected, 200 responses/hand, totaling 1600 responses for the entire experiment (800/hand). Reaction times of the button presses were collected by Presentation during the entirety of the EEG study.

After EEG administration, a survey was administered to evaluate judgments of groove, familiarity, and likability. Participants were asked to listen to the 20 songs (10 high groove, 10 low groove) presented during the EEG paradigm in 15-second clips. After each song, participants made judgments on what they heard. Using 7-point Likert scales, participants answered the following questions: (1) “How groovy was this song?” (2) “How much did you enjoy the song?” and (3) “How familiar were you with this song?” This portion of the experiment was also administered using the software Presentation over ER-3 ear inserts (Etymotic Research, Elk Grove Village, IL).

#### 2.1.5 Data analysis

EEG data for both experiments were processed in MATLAB 2017a using ERPLAB (Lopez-Calderon & Luck, 2014) and EEGLAB (Delorme & Makeig, 2004), both open-source software modules for MATLAB. First, data were referenced to the average of the left and right mastoids and were band-pass filtered from 0.05 to 100 Hz half-amplitude cutoff (-6 dB) using a Butterworth infinite impulse response (IIR) filter with a roll-off of 12 dB/octave. If any channels that are not pertinent to LRP analysis (i.e., not channels C3 or C4) were especially noisy, they were topographically interpolated using EEGLAB. Then, EEGLAB’s independent component analysis (ICA) was employed to identify and remove muscle and eye movement artifact. To make separate grand average waveforms of each stimulus category, each participant’s continuous EEG recording was truncated into 65-second sound trials and appended into separate stimulus categories (i.e., high groove, low groove, silent, noise). Next, using ERPLAB’s bin descriptor function, 1000 ms epochs were created while removing any epochs with incorrect responses; responses during stimulus presentation; and responses >1200 ms relative to the stimulus onset (i.e., only correct responses). Manual artifact rejection was then used to remove any epoch where a blink occurred during the LRP. Finally, in ERPLAB, average waveforms for contralateral and ipsilateral responses from electrodes C3 and C4 were generated with a stimulus-locked LRPs (S-LRP) baseline -200 to 0 ms, relative to the visual stimulus onset, and a response-locked LRPs (R-LRP) baseline of -800 to -600 ms, relative to the physical response. Using the Coles method calculation (Coles, 1989), S-LRP and R-LRP contralateral minus ipsilateral difference waves were created, resulting in eight averaged LRPs per participant: four S-LRPs per sound condition and four R-LRPs per sound condition. Because of the low signal-to-noise ratio of LRP difference waves, a 15 Hz low-pass half-amplitude (-6 dB) Butterworth IIR filter (roll-off of 12 dB/octave) was applied to averaged LRPs when measuring LRP onset latency, a common practice with LRPs (Kappenman et al., 2016). Mean amplitude was measured on LRPs within the original band-pass filtered range of 0.05–100 Hz. As determined by simulations using the jackknife technique, LRP onsets were measured as the time point corresponding to 50% of the peak amplitude (Miller et al., 1998). LRP amplitude was calculated as the mean amplitude within the measurement windows listed above relative to the baseline voltage.

#### 2.1.6 Statistical analyses

All statistical analyses were conducted using JASP (Version 0.18.3). LRP waveforms were statistically analyzed using both frequentist and Bayesian one-way repeated measures ANOVAs: four stimulus conditions (high groove, low groove, noise, silence) are labeled as independent variables and each LRP measure (stimulus-locked (S-LRP) onset, S-LRP amplitude, response-locked (R-LRP) onset, and R-LRP amplitude) labeled as the dependent variables. Frequentist ANOVAs with Greenhouse–Geisser corrections for sphericity violations are reported because they were performed as a first set of planned analyses, and because they provide effect sizes that are useful for future meta-analyses.

After observing null findings, we also performed Bayesian analyses to evaluate the strength of both null and alternative hypotheses. A Bayes Factor (BF) in support of the null hypothesis (BF_01_) or the non-null hypothesis (BF_10_) is reported for each analysis, and interpreted as follows: BF > 1 is “anecdotal evidence”; BF > 3 is “some evidence”; BF > 10 is “strong evidence”; and BF > 30 is “very strong evidence” (Rouder et al., 2009; Schmalz et al., 2023). Importantly, BF_10_ and BF_01_ are simply inverses of each other, that is, BF_10_ = 1/BF_01_, such that when a very small BF_10_ is shown in one of our tables, this implies that the BF_01_ would be fairly large. We used the r-scale fixed effect = 0.5, r scale random effect = 1.0, and r-scale covariate = 0.354 as coefficient prior (Andraszewicz et al., 2015), and the Model Prior was set to Uniform. We elected to Enforce the Principle of Marginality for fixed effects. We selected Automatic for Numerical Accuracy (uses 10,000 steps to approximate the integral for the Bayes factor), Automatic Integration Method (for approximating the marginal likelihood), and Automatic Posterior Samples (10,000 Markov Chain Monte Carlo samples to calculate the posterior and error %). These are all default parameters in JASP for Bayesian repeated-measures ANOVAs. The BF values represent the strength of evidence for the alternative hypothesis relative to the null or vice versa, which allows us to avoid dichotomized decisions (such as p-values in frequentist statistics) to accept/reject the hypothesis (Pek & Van Zandt, 2019). Correction for multiple comparisons (e.g., the simple correlations reported below) was deemed not necessary, consistent with recommendations for Bayesian analysis (Sjölander & Vansteelandt, 2019). Pearson’s correlations (using a stretched beta prior width = 1.0 to specify the expected plausible range effects before observing the data, alternative hypothesis = correlated) were also calculated, resulting in accompanying BF_01_ or BF_10_ values.

### 2.2 Results and discussion

Behavioral results indicate some evidence in favor of a lack of difference between stimulus conditions for median RT (*F*_3,60_ = 0.66, *p* = 0.552, *ɳ_p_^2^* = 0.032, BF_01_ = 7.69), but no evidence in favor of either the null or alternative hypothesis for the number of correct trials (*F*_3,60_ = 2.92, *p* = 0.054, *ɳ_p_^2^* = 0.127, BF_01_ = 0.75). This indicates equivalent sensory-motor performance on the primary task and, therefore, any observed difference in brain activity can be attributed to the manipulation of interest, rather than task difficulty. See Supplementary Figure S1 for reaction time and percentage correct data for Experiment 1. Groove ratings for the high-groove stimuli were higher than for the low-groove stimuli (*t*(20) = 9.23, *p* < .001, *d* = 2.01, BF_10_ = 1.06 × 10^6^), replicating Janata et al. (2012) and validating these categories for use in the LRP analyses below. As was found by Janata et al. (2012), there were moderate-to-large correlations between groove ratings and likability ratings for both HG (*r*(19) = 0.737, *p* < .001, BF_10_ = 233.60) and LG (*r*(19) = 0.624, *p* = .002, BF_10_ = 19.50) listening conditions; however, there was no evidence for relationships between groove ratings and familiarity ratings for both HG (*r*(19) = 0.068, *p *= .769, BF_10_ = 0.28) and LG (*r*(19) = 0.294, *p *= .196, BF_10_ = 0.59). The lack of correlation between groove and familiarity could be due to the music being older and less familiar for our participants compared with the participants tested by Janata et al. (2012). See Supplementary Figure S2A for average groove, likability, and familiarity ratings for Experiment 1.

**Fig. 1.**
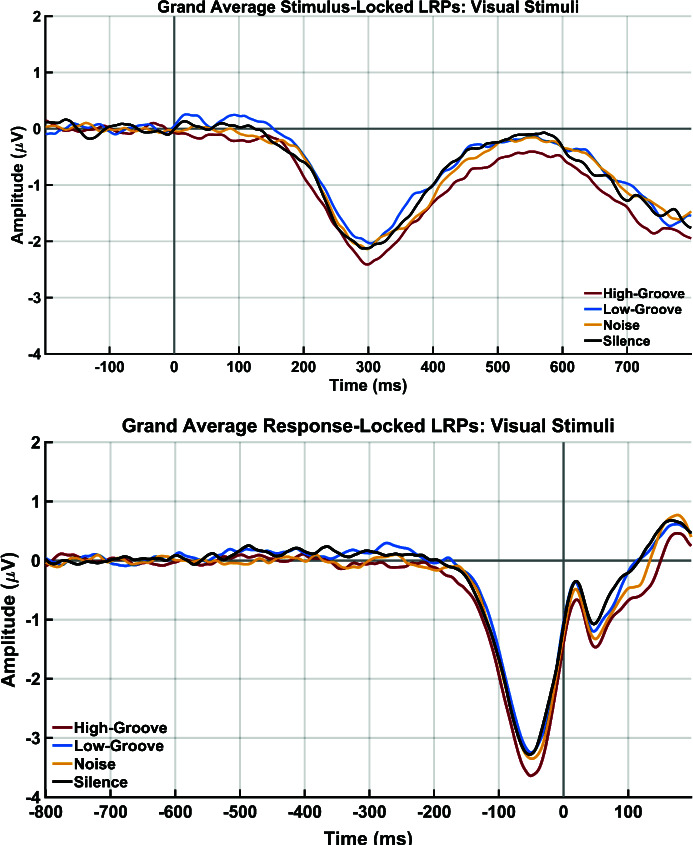
Experiment 1 grand averaged stimulus-locked lateralized readiness potentials (top) and response-locked lateralized readiness potentials for the high-groove, low-groove, noise, and silence conditions.

Figure 1 shows overlaid grand average waveforms across participants for each stimulus condition. The stimulus-locked LRPs are shown as emerging in all conditions just before 200 ms post-stimulus, consistent with them being elicited by the onset of the visual stimuli. In contrast, the response-locked LRPs emerge starting around 200 ms prior to the button press, consistent with their reflecting motor preparation and execution of the button press. Despite clear LRP responses and relatively good signal-to-noise ratio, these plots show a lack of difference in the latencies and amplitudes between all four conditions. This is substantiated by the statistical analyses with no differences between stimulus conditions for the S-LRP onset latency (*F*_3,60_ = 0.55, *p* = 0.567, *ɳ_p_^2^* = 0.027, BF_01_ = 8.47) and R-LRP onset latency (*F*_3,60_ = 0.396, *p* = 0.701, *ɳ_p_^2^* = 0.019, BF_01_ = 10.28). Similarly, there was also evidence for a lack of differences between stimulus conditions for the S-LRP mean amplitude (*F*_3,60_ = 1.86, *p* = 0.167, *ɳ_p_^2^* = 0.085, BF_01_ = 2.23) and the R-LRP mean amplitude (*F*_3,60_ = 1.10, *p* = 0.342, *ɳ_p_^2^* = 0.052, BF_01_ = 4.90).

Relationships between neural and behavioral data were also examined using Pearson’s correlations. Results show positive correlations with substantial evidence for the alternative hypothesis between mean S-LRP onset latency and median RT during HG listening (*r*(19) = .64, *p* = 0.002, BF_10_ = 26.89) and between mean S-LRP onset latency and median RT during LG listening (*r*(19)* = *.61, *p* = 0.003, BF_10_ = 14.83). These correlations indicate that with increased mean S-LRP onset latency, there was increased median RT, consistent with the LRPs reflecting overall motor performance. There were no other strong correlations between LRP latency and reaction time. There were also no strong correlations between LRP mean amplitude and median RT for any stimulus conditions. Relationships between R-LRP measures and median RT were not calculated because they are related: R-LRP epochs are based upon when responses occur (i.e., RT) and, therefore, are highly correlated. Correlations between mean neural measures and mean subjective song rating showed no strong relationships between any of the ERP measures and their corresponding subjective groove ratings, consistent with lack of evidence for high-groove songs to modulate ERP activity compared with low-groove songs or the control conditions.

The results of Experiment 1 failed to show any enhanced amplitude of the LRP while listening to high-groove music or any difference between any of the four conditions, and in fact showed some evidence in favor of the null hypothesis. This could indicate that listening to high-groove music does not influence primary motor cortical activity. It is also possible, however, that ignoring the music in order to perform the primary visuo-motor task that evokes the LRP limits the engagement of the motor system that would normally occur when participants listen to high-groove music in more naturalistic conditions. We, therefore, conducted a second experiment that required participants to pay attention to the music by detecting changes of intensity in the music itself.

## 3 Experiment 2

### 3.1 Methods

#### 3.1.1 Participants

For Experiment 2, 48 participants were originally recruited. Three participants were excluded due to pre-existing medical conditions, eight participants were excluded due to study incompletion, one participant was excluded for poor behavioral performance, two participants were excluded for having hearing deficiencies, and three participants were excluded due to equipment issues. The final 31 participants were between the ages of 18 and 39 years (*M* = 21.9 years, *SD* = 4.5 years). All but one participant was right handed. Eighteen participants self-reported having musical experience (of those people, age start: *M* = 10.33 years old, *SD* = 3.01 years old, range = 6–18 years old; years of practice: *M* = 4.56 years, *SD* = 2.31 years, range = 1–10 years) and nine participants self-reported having dance experience (of those people, age start: *M* = 10.78 years old, *SD* = 5.83 years old, range = 4–19 years old; mean years of practice: *M* = 3.44 years, *SD* = 2.01 years, range = 2–8 years). Of these participants, five had both music and dance experience. Ten participants did not have any musical or dance experience.

#### 3.1.2 Stimuli

The stimuli were the same as in Experiment 1, except as follows. Instead of visual stimuli, contralateral button presses were indicated by changes in brief acoustic intensity changes embedded into the sound files using MATLAB 2016b (The Mathworks, Natick, MA, USA). High-groove songs, low-groove songs, and noise conditions were embedded with 20 increasing and 20 decreasing volume changes each 300 ms in duration with a 30 ms onset and offset ramp to ensure a smooth transition between volume changes. For silent conditions, white noise was presented either loudly or softly at the same intensity as the other conditions for the same duration and frequency. Loud conditions were ~15 dB increase (~75 dB) from the baseline volume (~60 dB); soft conditions were ~15 dB decrease (~45 dB) from the baseline volume. In between response trials, a fixation cross, also in white on a black background (measuring at this visual angle), was visible to limit lateral eye movements. The monitor was viewed at a distance of 42 inches. No additional visual stimuli were presented during this experiment.

#### 3.1.3 EEG setup and procedure

The EEG setup was the same as in Experiment 1. The procedure was the same as Experiment 1 except it used auditory-only stimuli created for Experiment 2, and adapted the Presentation code from Experiment 1. Participants listened actively to the music and reported whether they heard increases or decreases in intensity of the music by pressing the same buttons as in Experiment 1. The volume changes used to elicit the LRPs were presented slightly differently. The inter-stimulus interval (ISI) between volume changes was pseudo-randomized between 1000, 1100, 1200, and 1300 ms, and presented randomly with an even distribution. Increases and decreases in volume, instead of visual symbols, were assigned to either right- or left-hand button presses.

#### 3.1.4 Data and statistical analysis

Experiment 2 data processing and statistical analyses were identical to Experiment 1, except for some post hoc analyses as described in the Results section.

### 3.2 Results and discussion

Behavioral results show evidence in favor of a difference between stimulus conditions for median RT (*F*_3,90_ = 16.70, *p* < 0.001, *ɳ_p_^2^* = 0.358, BF_10_ = 1.02 × 10^6^). However, this was mainly due to shorter RT for the noise condition; when only the HG and LG conditions were included, there was no indication of difference between those two critical conditions (*F*_1,30_ = 2.46, *p* = 0.127, *ɳ_p_^2^* = 0.076, BF_10_ = 0.682). There was also substantial evidence for differences between conditions in percentage correct (*F*_3,90_ = 42.83, *p*<.001, *ɳ_p_^2^* = .588, BF_10_ = 1.84 × 10^14^), and this was also true when just including the HG and LG conditions (*F*_1,30_ = 20.93, *p*<.001, *ɳ_p_^2^* = .411, BF_10_ = 241.06), with the worst performance for high-groove songs, and increasingly better for performance for low-groove, silence, and noise conditions in that order. See Supplementary Figure S3 for reaction time and percentage correct data for Experiment 2. Groove ratings for the high-groove stimuli were higher than for the low-groove stimuli (*t*(29) = 12.46, *p *< .001, *d* = 2.28, BF_10_ = 2.11 × 10^10^). There were moderate-to-large correlations between groove ratings and likability ratings for both HG (*r*(28) = 0.851, *p *< .001, BF_10_ = 4.72 × 10^6^) and LG (*r*(28) = 0.484, *p *= .007, BF_10_ = 7.53) listening conditions. There was also evidence for a relationship between groove ratings and familiarity ratings for HG (*r*(28) = 0.591, *p *< .001, BF_10_ = 63.58) but not for LG (*r*(28) = 0.229, *p *= .224, BF_10_ = 0.46). See Supplementary Figure S2B for average groove, likability, and familiarity ratings for Experiment 2.

**Fig. 2.**
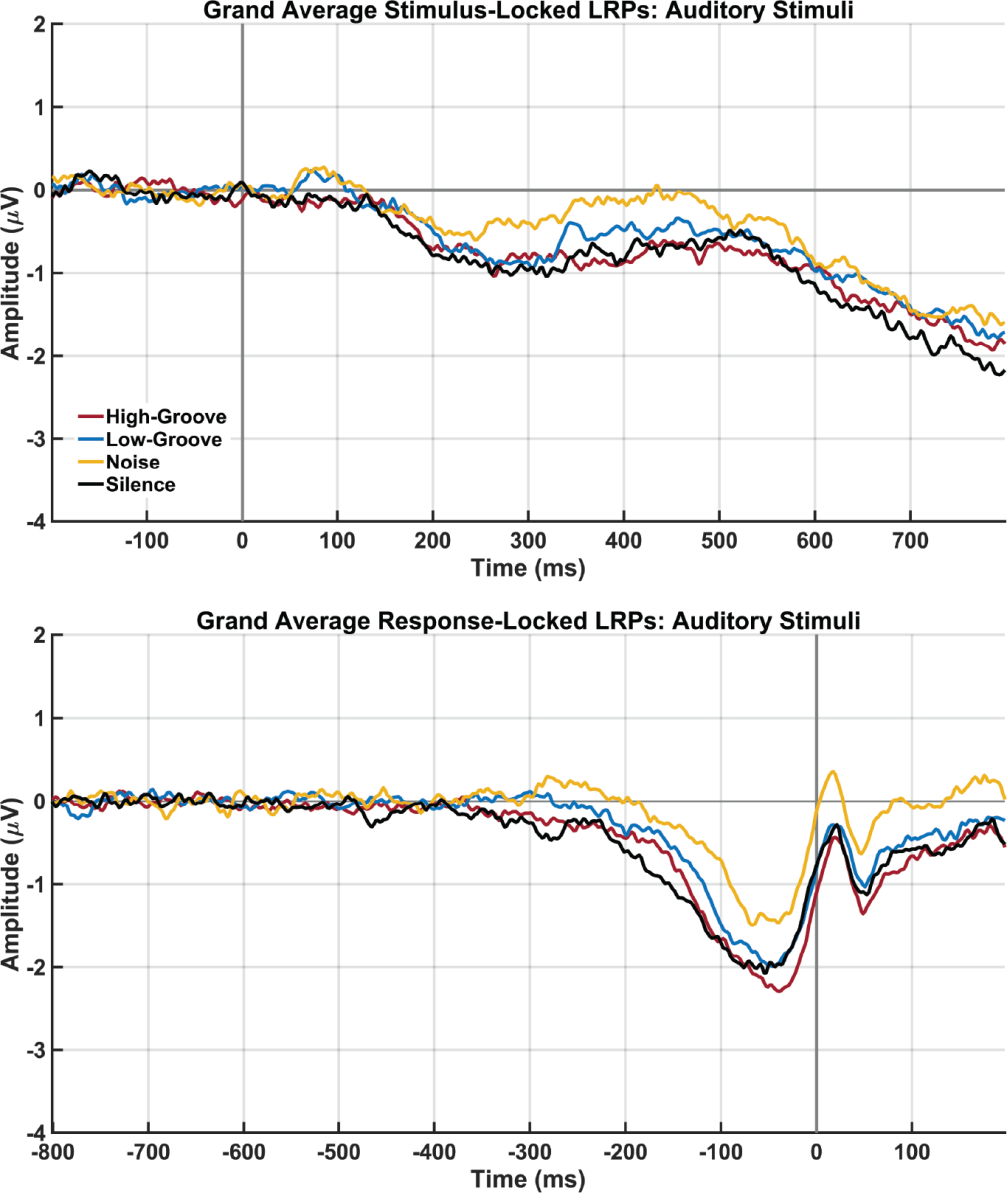
Experiment 2 grand averaged stimulus-locked lateralized readiness potentials (top) and response-locked lateralized readiness potentials for the high-groove, low-groove, noise, and silence conditions.

Figure 2 shows overlaid grand average waveforms across participants for each stimulus condition. The waveforms generally show more baseline noise than in Experiment 1, though this noise amplitude is still much smaller than LRP responses. The increased noise may be due to the recordings being performed in a different building than in Experiment 1, though with the same EEG system. It is also apparent that the S-LRP responses have a different morphology and smaller amplitudes than in Experiment 1, which we attribute to the use of less noticeable intensity changes in the music, rather than the symbols flashing on the screen that were used in Experiment 1. Results show that there is evidence for a lack of differences between stimulus conditions for the S-LRP onset latency (*F*_3,81_ = 1.48, *p *= .227, *ɳ_p_^2^* = .052, BF_01_ = 3.97) and R-LRP onset latency (*F*_3,81_ = 1.22, *p *= .309, *ɳ_p_^2^* = .042, BF_01_ = 5.35). Unlike in Experiment 1, however, there was substantial evidence for differences between stimulus conditions for the S-LRP mean amplitude (*F*_3, 81_ = 7.36, *p *< .001, *ɳ_p_^2^* = .197, BF_10_ = 132.06) and the R-LRP mean amplitude (*F*_3, 81_ = 11.03, *p* < 0.001, *ɳ_p_^2^* = 0.269, BF_10_ = 5326.61). However, this was mostly due to smaller LRP amplitudes in the noise condition, rather than any difference between the high-groove and low-groove conditions. When the high-groove and low-groove conditions were the only ones included, there was no evidence for difference between these two conditions for the S-LRP mean amplitude (*F*_3,90_ = 2.04, *p* = 0.163, *ɳ_p_^2^* = 0.064, BF_10_ = 0.62) and the R-LRP mean amplitude (*F*_3,90_ = 3.69, *p *= .110, *ɳ_p_^2^* = .110, BF_01_ = 1.14).

Relationships between neural and behavioral data were also examined using Pearson’s correlations. Results show positive correlations with substantial evidence for the alternative hypothesis between mean S-LRP onset latency and median RT during HG listening (*r*(28) *=* .50, *p* = 0.005, BF_10_ = 9.43) but not between mean S-LRP onset latency and median RT during LG listening (*r*(29) *=* .23, *p* = 0.223, BF_10_ = 0.454). There were no other strong correlations between LRP latency and reaction time. There were also no strong correlations between LRP mean amplitude and median RT for any stimulus conditions. Correlations between mean neural measures and mean subjective song rating showed no strong relationships between any of the ERP measures and their corresponding subjective groove ratings, consistent with lack of evidence for high-groove songs to modulate ERP activity compared with low-groove songs or the control conditions.

Despite participants paying more attention to the music in Experiment 2 than in Experiment 1, we did not find any compelling evidence for larger LRP responses during the high-groove music compared with the low-groove music. Thus, the results of the two experiments both fail to support the idea that listening to high-groove music enhances activity in primary motor cortex and are inconsistent with the findings of Stupacher et al. (2013).

## 4 Discussion

In two experiments, participants performed a simple button pressing task while listening to high-groove music, low-groove music, noise, or silence in the background (Experiment 1), or while actively attending to the auditory stimuli (Experiment 2). In both experiments, we found Bayesian evidence in favor of no difference between listening to high-groove vs. low-groove music on the amplitude or latency of the LRP response. These findings fail to support the theory that high-groove music engages the primary motor cortex—the main source of the LRP—more so than low-groove music, noise, or silence. Although the button press tasks clearly activated the motor cortex in both experiments, as reflected most clearly by the response-locked LRPs, the music or noise conditions did not further modulate the LRPs and thus may not facilitate neurons in the motor cortex (cf. Kappenman et al., 2016).

These results fail to provide convergent support for a prior study that measured motor-evoked potentials in response to TMS pulses to the hand and forearm regions of left primary motor cortex, while participants listened to high-groove and low-groove music (Stupacher et al., 2013). In that study, seven trained musicians showed larger motor-evoked potentials in the right hand and forearm muscles while listening high-groove music compared with low-groove music, after subtracting out responses during spectrally matched noise. The eight non-musically trained participants they tested, however, showed smaller responses during high-groove music than during low-groove music, leading to counter-intuitive findings in that group of participants. This was despite similar perception of groove in the musically trained and untrained groups. Given the small sample sizes, experimental design, and possibly unreliable motor-evoked potentials that were recorded (TMS pulses were set at a level that only produced a response half of the time in the absence of sound), it would be worthwhile performing a direct replication of this study with larger samples, a pre–post listening experimental design, and more intense TMS stimulation. Virtual-lesion studies using repetitive TMS of primary motor cortex could also be used to disrupt perception of groove, which would provide causal evidence that this brain area mediates groove perception. At present though, the current findings with LRPs and prior findings using TMS make it unclear whether listening to high-groove music modulates activity in primary motor cortex.

Besides the limitations of the Stupacher et al. (2013) study discussed above, it is also possible that the LRP technique used in the current study does not measure a comparable aspect of primary motor cortex activity compared with the TMS-evoked motor potentials used by Stupacher et al. (2013). TMS-evoked motor potentials index general motor excitability, whereas the LRP indexes motor preparatory activity prior to a cued movement. Although both types of activity could potentially be modulated by listening to high-groove music, this is not necessarily the case. For example, primary motor cortex activity during cued movements could in principle be insulated from irrelevant stimuli or tasks, such as background music. This would be particularly relevant to Experiment 1 of the current study in which the background music was not relevant to performing the visual-motor task that evoked the LRP. In Experiment 2, however, the music was no longer in the background and participants had to attend to intensity changes in order to perform the task. Even in the case of Experiment 2, though, it is still possible that the single button press type of movement used in an LRP paradigm is not ideal for revealing an influence of high-groove music because high-groove music is more likely to prime repetitive rhythmic movement that is synchronized with the beat of the music.

Another possible limitation of the current study is that the primary tasks used in the two experiments may interfere with the experience of groove and any associated modulation of activity in motor cortex. In particular, although the task in Experiment 1 was fairly easy, it required participants to at least partially ignore the music in the background, and this inattention to the music could have attenuated the experience of groove and any associated motor cortex facilitation. This motivated the design for Experiment 2, which required greater attention to the music in order to detect the intensity changes in the music itself. However, it remains a possibility that performing a task that is irrelevant to the rhythm and the experience of groove could have still attenuated the experience of groove, despite the active listening to the music. Perhaps if changes in volume were aligned with the musical beat, the feeling of “groove” might have increased during the LRP task. Thus, future studies should measure LRPs or other motor-related EEG responses like the contingent negative variation (CNV) while participants actively perform a task that requires rhythm or timing judgments (cf. Pfeuty et al., 2003; Snyder et al., 2011) while listening to high-groove vs. low-groove music to see whether this manipulation modulates motor activity specifically during a rhythm or timing task. It would also be worthwhile performing an LRP or CNV experiment in which participants make groove judgments immediately after listening to each stimulus, like in some past studies (Matthews et al., 2020; Zalta et al., 2024), in case such active thinking about groove is a prerequisite to modulating the motor cortex.

While a general motor theory of groove might predict the primary motor cortex as a mediator between music listening and wanting to move, more detailed theories and many datasets point to non-primary motor areas being more important for groove, and beat perception more generally (for reviews, see Proksch et al., 2020; Snyder et al., 2024; for a meta-analysis, see Gordon et al., 2018). For example, the Action Simulation for Auditory Prediction (ASAP) hypothesis proposes that motor planning regions in the frontal and parietal lobes simulate movement, which informs beat-based predictions sent to auditory cortical regions (Patel & Iversen, 2014). Similarly, predictive coding models of beat and groove perception state that premotor and supplementary motor areas learn beat-based models of rhythmic structure and provide predictions to auditory cortex to aid music listening (Cannon, 2021; Cannon & Patel, 2021; Vuust & Witek, 2014). Furthermore, the auditory cortex calculates prediction errors that are fed back to motor areas to update the models. This type of model proposes that groove results from a moderate amount of prediction errors while listening to syncopated music (Vuust & Witek, 2014; Vuust et al., 2018). Oscillator models similarly propose interactions between motor and auditory areas as the basis of beat perception and groove, but uniquely propose that both motor and auditory cortical areas entrain with the beat of music during listening (Large & Snyder, 2009; Harding et al., 2025; Zalta et al., 2024). Furthermore, recent oscillatory models propose that banks of oscillators tuned to different beat frequencies adjust their connection weights with each other as a learning mechanism (Tichko & Large, 2019; Zalta et al., 2024). This in turn enables model learning and the emergence of groove due to a moderate amount of mismatch between predictions and rhythmic input, similar to predictive coding models.

Given that these different models have much in common, it is difficult to evaluate which is better supported by existing data. Nevertheless, the data do strongly support the more general idea that beat and groove perception result from activity in premotor and supplementary motor areas, in addition to auditory cortex. For example, fMRI studies find activations in premotor and supplementary cortical areas, auditory cortex, cerebellum, and striatal areas during reproduction of rhythms or synchronization to the beat of rhythms (Chen et al., 2005, 2006; Chen et al., 2008b; Grahn & Brett, 2007), and also while listening to rhythms without actually moving (Chen et al., 2008a; Grahn & McAuley, 2009; Grahn & Rowe, 2009; Schubotz et al., 2000; Schubotz & von Cramon, 2001). There is also one fMRI study showing greater activity during moderate complexity, high-groove patterns in brain areas linked to beat perception and reward processing, including parts of the basal ganglia and the supplementary motor area (Matthews et al., 2020).

An EEG study on patients with damage to basal ganglia and cerebellum showed reduced neural tracking of the beat of rhythms in auditory cortex (Nozaradan et al., 2017), providing further evidence for the importance of these subcortical motor areas in beat processing. EEG and MEG studies on healthy participants also provide further evidence of motor activation during beat-based processing (Cheng et al., 2022; Fujioka et al., 2012; Iversen et al., 2009; Large & Snyder, 2009; Snyder et al., 2011), though the limited spatial resolution of these techniques can make it less certain whether activity comes from premotor, supplementary motor, or primary motor cortex. Finally, brain stimulation studies provide evidence for important roles for beat perception of right dorsal premotor cortex (Lazzari et al., 2025), and the dorsal audio-motor pathway in the parietal lobe (Ross et al., 2018). As described earlier, there is also one TMS study providing evidence for high-groove music activating the primary motor cortex more than low-groove music, though this finding was limited to musicians (Stupacher et al., 2013). Finally, a recent TMS study found that inhibitory stimulation to left supplementary motor area increased the urge to move (but not feelings of pleasure) while listening to relatively complex instrumental music clips (Spiech et al., 2025). Thus, other than one TMS study on groove (Stupacher et al., 2013), the bulk of evidence from a variety of brain methods on beat perception and groove does not implicate primary motor cortex as an important part of the brain network that mediates beat perception and groove. This overall pattern of findings in the literature is consistent with the lack of evidence for high-groove music activating primary motor cortex more than low-groove music in the current study. Across all the TMS studies, however, the effect sizes are relatively modest, and there are also inconsistent findings across studies. For example, Ross et al. (2018) obtained null results for stimulation of the supplementary motor area, and Lazzari et al. (2025) obtained null results for stimulating supplementary motor area and left premotor cortex in Lazzari et al. (2025), despite these areas being implicated for beat and/or groove perception by other studies using TMS, fMRI, EEG, and or/ MEG. Thus, the small and inconsistent findings, plus the use of different paradigms, demonstrate the need for additional TMS studies on beat perception and groove, including both direct and conceptual replications (Nosek & Errington, 2017).

### 4.1 Summary

In conclusion, we found lack of evidence for modulation of primary motor cortex activity while listening to high-groove music compared with listening to low-groove music, as measured with the lateralized readiness potential. This partially contradicts a previous brain stimulation study, which found larger motor-evoked potentials while participants listened to high-groove music than when participants listened to low-groove music (Stupacher et al., 2013). Future studies should replicate our findings and the Stupacher et al. (2013) findings, as well as test for neural correlates of groove in a wider variety of brain areas, including auditory cortex, premotor cortex, and the basal ganglia.

## Supplementary Material

Supplementary Material

## Data Availability

The data are not available because ethics approval was not obtained for making the data available. Stimuli, presentation materials, and analysis scripts are given in this paper’s project page on the Open Science Framework at https://osf.io/fyq8d/.
