# Supplementary material for: Musical groove listening does not enhance primary motor cortex activation
Source: Imaging Neurosci (Camb). 2026 Apr 3;4:IMAG.a.1185. doi: 10.1162/IMAG.a.1185 (PMC13051682; doi:10.1162/IMAG.a.1185)
Supplement: Supplementary Material [file IMAG.a.1185_supp.pdf]

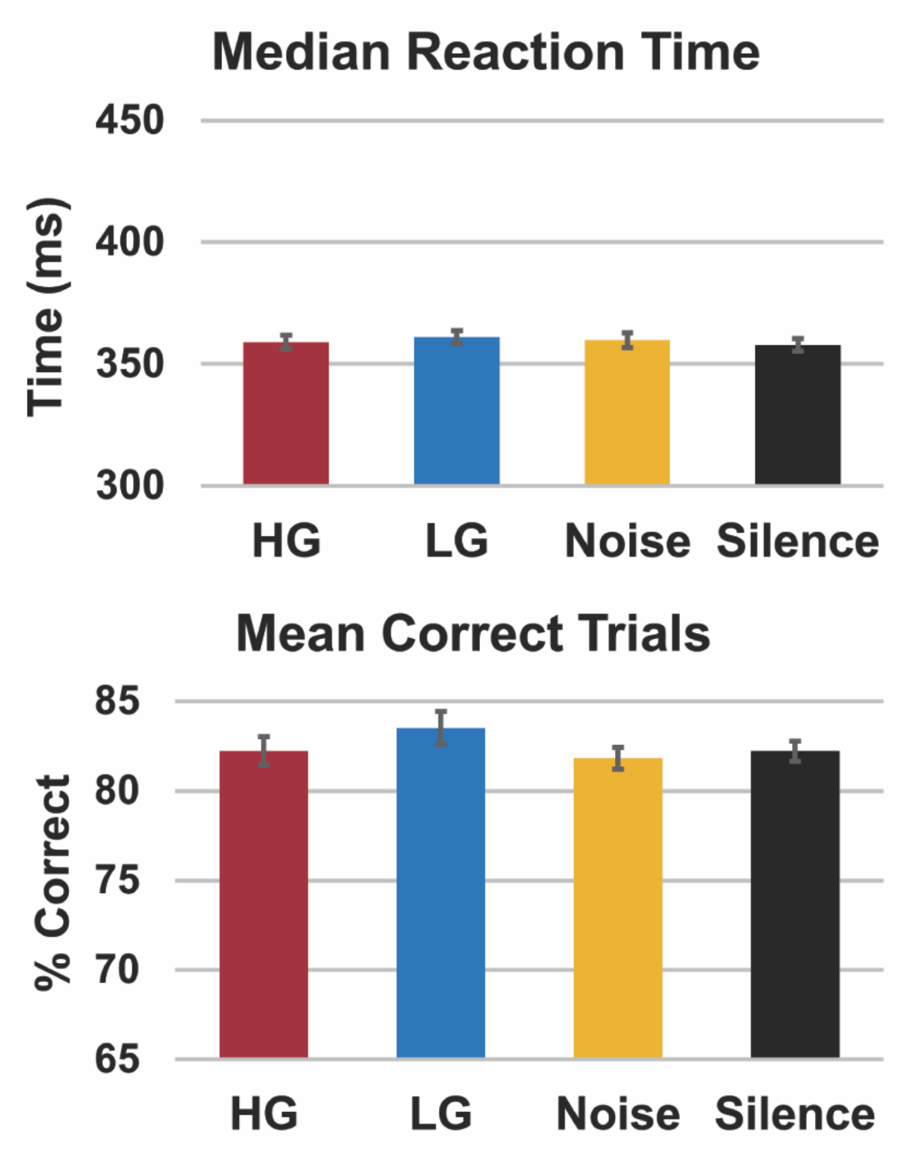

Figure S1. Median reaction time (top) and % Correct for Experiment 1 on the visuo-motor task.

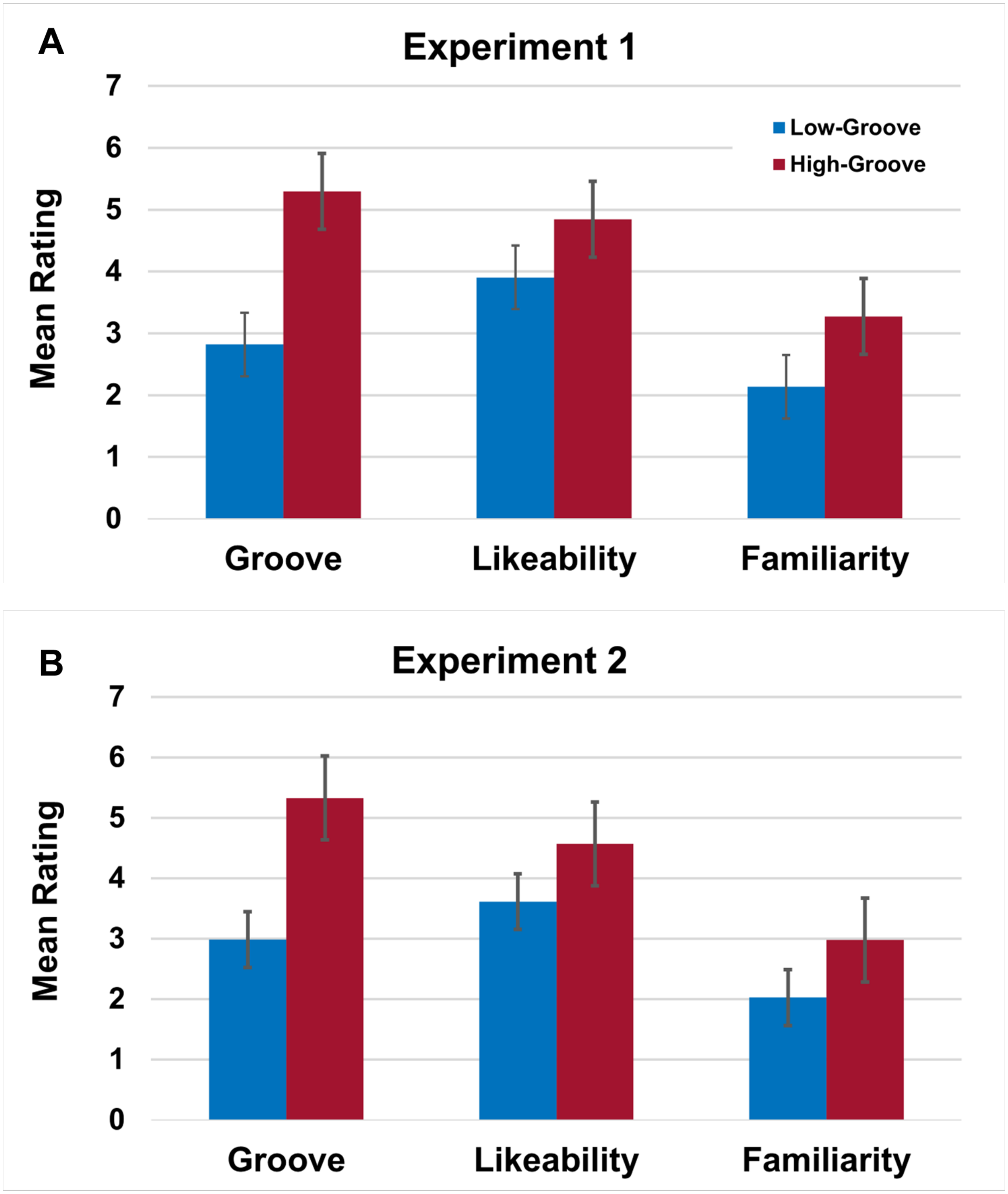

Figure S2. Average Groove, Likeability, and Familiarity Ratings for Experiment 1 (A) and Experiment 2 (B).

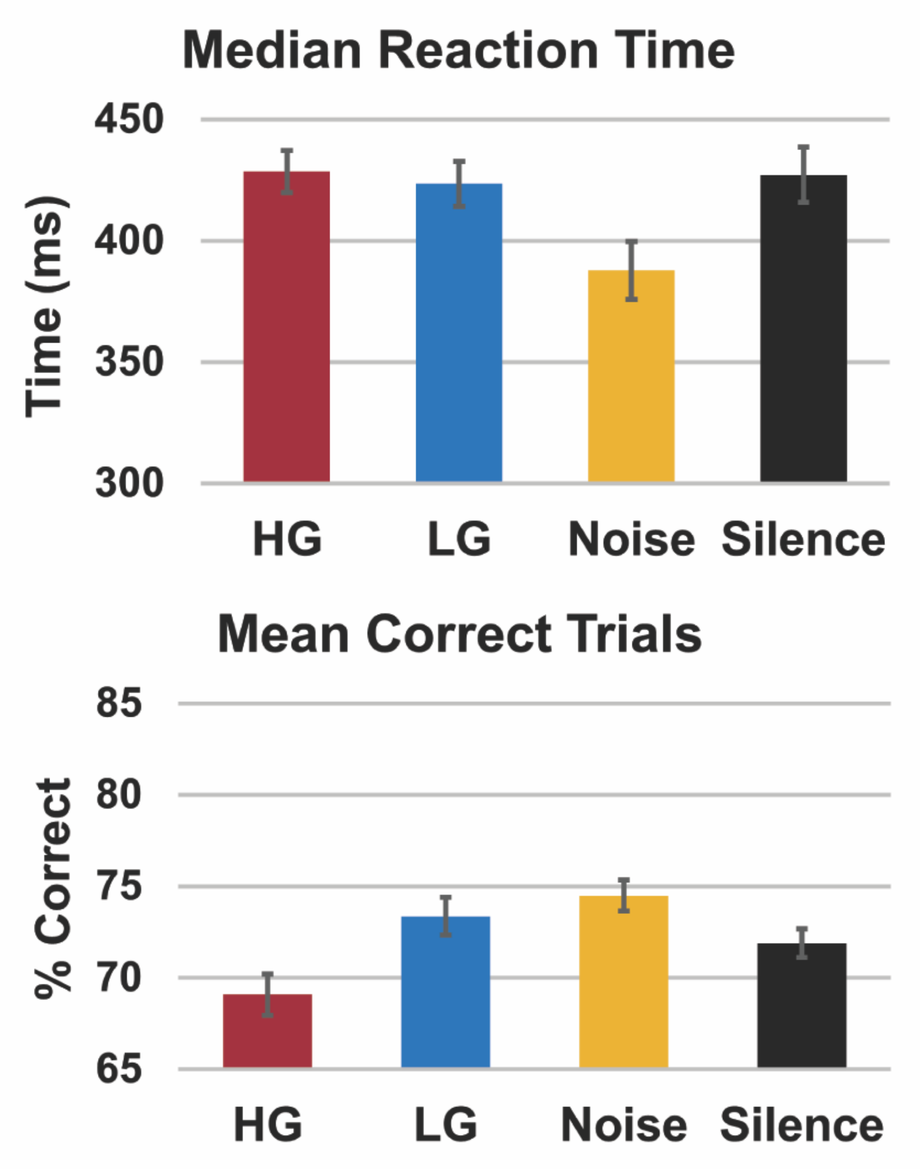

Figure S3. Median reaction time (top) and % Correct for Experiment 2 on the audio-motor task.
